# Miller Fisher syndrome following BNT162b2 mRNA coronavirus 2019 vaccination

**DOI:** 10.1186/s12883-021-02489-x

**Published:** 2021-11-18

**Authors:** Yamato Nishiguchi, Hirofumi Matsuyama, Kuniko Maeda, Akihiro Shindo, Hidekazu Tomimoto

**Affiliations:** 1 Department of Neurology, Graduate School of Medicine, Mie University, Tsu, Mie 514-8507 Japan; 2 Department of Ophthalmology, Mie Prefectural Shima Hospital, Shima, Mie 517-0595 Japan

**Keywords:** Miller fisher syndrome, Guillain–Barré syndrome, SARS-CoV-2, COVID-19, Vaccination

## Abstract

**Background:**

The coronavirus disease 2019 (COVID-19) pandemic, caused by severe acute respiratory syndrome coronavirus 2 (SARS-CoV-2), began in late 2019. One of the vaccines approved against COVID-19 is the BNT162b2 mRNA COVID-19 vaccine (Pfizer/BioNTech).

**Case presentation:**

We present the case of a 71-year-old man with no history of the SARS-CoV-2 infection or any recent viral or bacterial illnesses who presented with bilateral oculomotor palsy and limb ataxia after BNT162b2 mRNA COVID-19 vaccination. The diagnosis of Miller Fisher syndrome (MFS) was established based on physical examination, brain magnetic resonance imaging (MRI), cerebrospinal fluid analysis (CSF), and positron emission tomography (PET). There was no evidence of other predisposing infectious or autoimmune factors, and the period from COVID-19 vaccination to the appearance of neurological symptoms was similar to that of other vaccines and preceding events, such as infection.

**Conclusion:**

Guillain–Barré syndrome (GBS) and its variants after COVID-19 vaccination are extremely rare. Note that more research is needed to establish an association between MFS and COVID-19 vaccines. In our opinion, the benefits of COVID-19 vaccination largely outweigh its risks.

## Background

The coronavirus disease 2019 (COVID-19) pandemic, caused by severe acute respiratory syndrome coronavirus 2 (SARS-CoV-2), began in late 2019. Several vaccines, including BNT162b2 mRNA COVID-19 vaccine (Pfizer/BioNTech), were developed and have been shown to be effective worldwide. Although vaccination is the best way to control the pandemic, the neurological complications have not been fully elucidated. The Guillain–Barré syndrome (GBS) is a peripheral immune-mediated neuropathy causing muscle weakness, sensory disturbances, and dysautonomia and often involves cranial neuropathies. Post-vaccination GBS has been reported recently [1]. In this paper, we study the case of the Miller Fisher syndrome (MFS) recorded after receiving the first dose of BNT162b2 mRNA COVID-19 vaccine. We also reviewed all previous cases of GBS and its subtypes that occurred after COVID-19 vaccination.

## Case presentation

**Fig. 1:**
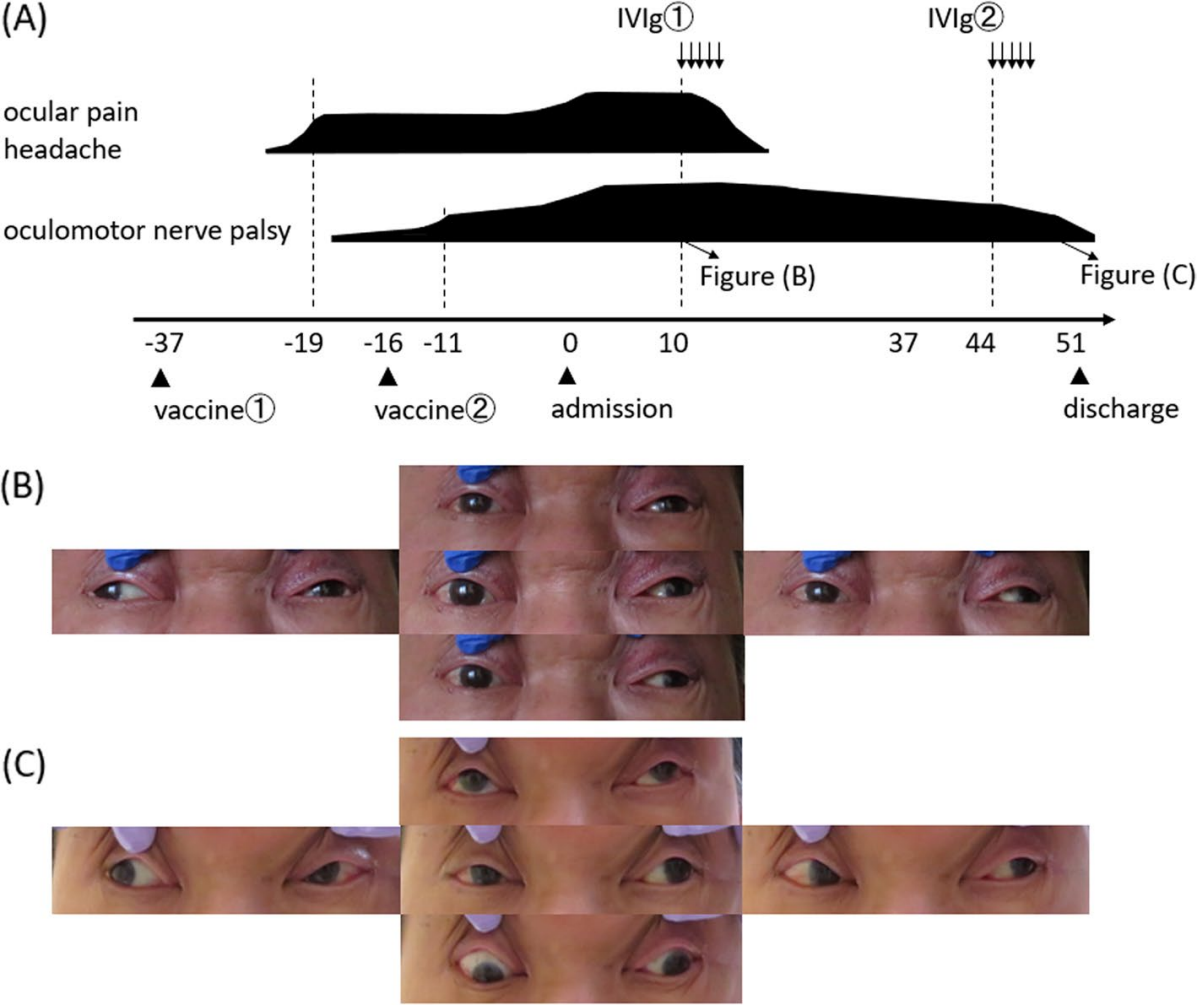
**A** Timeline depicting patient admission, examination, and treatment. For further details, refer to the main text. **B** Eye movement before treatment (on the 10th hospital day). **C** Eye movement after intravenous immunoglobulin treatment (on the 41st hospital day)

A 71-year-old man with a medical history of diabetes mellitus and diabetic ophthalmoplegia (7 years ago, and recovered completely) presented with headache and ocular pain 18 days after the first dose of BNT162b2 mRNA COVID-19 vaccine. The patient’s symptoms progressed over the next 8 days to ptosis and ocular motility disorder. He took the second dose 3 weeks after the first vaccination, and his neurological symptoms continued to worsen after the second dose. On physical examination on admission (16 days after the second dose), the patient was vitally stable, afebrile, and on room air without signs of respiratory distress. Neurological examination showed bilateral ptosis, bilateral pupillary light reflex disappearance, oculomotor nerve palsy (Fig. 1), left-dominant limb ataxia, and mild ataxic gait.

The muscle strength of the limbs was normal, and the tendon reflex was not reduced. Complete blood cell count and coagulation profile were normal. Hemoglobin A1c was 7.1%. CEA, CA19–9, IL-2 receptor, ACE, lysozyme, IgG4, MPO-ANCA, PR3-ANCA, antinuclear antibody, anti-SS-A antibody, anti-SS-B antibody, and anti-DNA antibody were negative. A lumbar puncture was performed, and the consequent cerebrospinal fluid (CSF) analysis showed normal white cell counts (1 cell/microliter) and elevated protein at 67 mg/L (reference range, 15–40 mg/L). The gram staining, culture, and cytology analysis of the CSF were negative. Brain magnetic resonance imaging (MRI) was normal except for minor venous dilation of the middle cranial fossa. Moreover, thin slice brain stem and the gadolinium-enhanced MRI showed no lesions with enhancing effects. Magnetic resonance angiography and venography were normal. Anti-ganglioside antibodies (i.e., GM1, GM2, GM3, GD1a, GD1b, GD3, GT1a, GT1b, GQ1b, Gal-C, and GT1a) in the serum were examined, and all tests resulted negative. Nerve conduction study of the patient’s limbs showed normal findings. Positron emission tomography/computed tomography (PET/CT) did not suggest the presence of malignant tumors or inflammatory diseases. Incomplete MFS was diagnosed because other diseases, such as meningeal carcinomatosis, hypertrophic dural inflammation, infection, collagen disease, cavernous sinus fistula, and the Tolosa–Hunt syndrome, which had similar symptoms, were ruled out. The patient received an intravenous immunoglobulin (IVIg) treatment. No complications were observed during or after the treatment. The patient’s headache and eye pain improved after five doses of IVIg. Moreover, the light reflex, ptosis, ophthalmoplegia, limb ataxia, and ataxic gait gradually improved after the second course of IVIg was administered. All symptoms were improved completely after 1 month, and no recurrence was observed.

## Discussion

This case report revealed two important clinical issues. First—MFS can occur following BNT162b2 mRNA COVID-19 vaccination, and second—the peak of the symptoms may be delayed in the common form of MFS, and the anti-ganglioside antibodies were all negative.

MFS is a rare variant of GBS, which is characterized by the acute onset of external ophthalmoplegia, ataxia, and loss of tendon reflexes. There exist incomplete forms of MFS, including acute ataxic neuropathy (which can be diagnosed in the absence of ophthalmoplegia) and acute ophthalmoparesis (which may occur in the absence of ataxia) [2]. MFS has been recorded to be preceded by infections similar to those preceding GBS, and in some instances, vaccination. Symptoms develop within 1–4 weeks after the antecedent cause [3]. In several reports, GBS was linked with a few vaccines, namely, those against rabies, hepatitis A and B, polio, and influenza [4]. However, to our knowledge, MFS has not been reported to be associated with COVID-19 vaccination. Therefore, we investigated not only MFS but also GBS and its variant after COVID vaccination. We systematically searched in the search engine PubMed using the keywords “COVID- 19” or “SARS-CoV-2”, “vaccination” or “vaccine”, and “Guillain–Barré syndrome”, “Miller Fisher syndrome”, “acute ataxic neuropathy”, “acute ophthalmoparesis”, “pharyngeal cervical brachial”, “polyneuritis cranialis”, “bilateral facial weakness with paresthesia”, “acute inflammatory demyelinating polyneuropathy”, “acute motor axonal neuropathy” or “acute motor sensory axonal neuropathy” to identify all studies from December 01, 2019 to July 30, 2021. Thirty-four articles were found. Further, we selected all cases in which GBS and its variants were detected after COVID-19 vaccination. Cases that were concluded to have a negative causal relationship with COVID-19 vaccination were excluded. Consequently, 24 cases were analyzed (Table 1).

**Table.1:** Clinical characteristics of published cases of Guillain–Barré syndrome/Miller Fisher syndrome following COVID-19 vaccination

Case No.	Age, Sex	Symptoms limb weakness	Paraesthesia	FD	OP	RF	Other neurological signs	Diagnosis	AGA	Comorbidies, Underlying disease	Treatment	Vaccine type	Time to symptom onset	Reference No.
1	54,M		+	+				GBS	negative	nothing	oral PSL	Oxford–AstraZeneca	12 days	[5]
2	20,M		+	+			headache	GBS	negative	ulcerative colitis	oral PSL	Oxford–AstraZeneca	21 days	[5]
3	57,M	+	+	+	+		dysarthria	GBS	negative	asthma, osteoarthritis	IVIg	Oxford–AstraZeneca	11 days	[5]
4	55,M		+	+				GBS	negative	HTN	nothing	Oxford–AstraZeneca	22 days	[5]
5	66,M		+	+			tongue numbness, ataxic gait	BFP	negative	n.d.	IVIg	Oxford–AstraZeneca	7 days	[1]
6	43,M			+			neck pain, urinary retention,dysphagia	BFP	negative	n.d.	IVIg	Oxford–AstraZeneca	11 days	[1]
7	51,M		+	+			leg pain	BFP	GM3(+)	n.d.	nothing	Oxford–AstraZeneca	7 days	[1]
8	71,F	+		+			back pain, abdominal pain, altered taste	BFP	negative	COVID-19 (47 days ago)	nothing	Oxford–AstraZeneca	12 days	[1]
9	53,M		+	+			back pain	BFP	n.d.	n.d.	nothing	Oxford–AstraZeneca	8 days	[1]
10	32,M		+	+			dysphagia, headache, dysarthria	GBS	n.d.	GBS (14 years ago)	IVIg, PP	vector-based vaccine	8 days	[6]
11	62,F	+	+			+	dysarthria, dysphagia	GBS	n.d.	bronchiectasis, asthma, osteoporosis, migraine	IVIg, MV	Oxford–AstraZeneca	8 days	[7]
12	74,M	+		+			back pain, urinary retention, dysarthria	AIDP, ITP	n.d.	HTN, gout, HL, cardiomyopathy	IVIg, PP, PSL	Moderna	13 days	[8]
13	43,F	+		+		+	back pain	GBS	n.d.	n.d.	IVIg, MV	Oxford–AstraZeneca	10 days	[9]
14	67,F	+	+	+		+	dysphagia	GBS	negative	n.d.	IVIg, PP, MV	Oxford–AstraZeneca	14 days	[9]
15	53,F	+	+	+		+	tongue numbness, back pain	GBS	negative	n.d.	IVIg, MV	Oxford–AstraZeneca	12 days	[9]
16	68,F	+	+	+		+	dysphagia, facial numbness	GBS	negative	n.d.	IVIg, MV	Oxford–AstraZeneca	14 days	[9]
17	70,M		+	+		+	bulbar palsy	GBS	n.d.	n.d.	IVIg, MV	Oxford–AstraZeneca	11 days	[9]
18	69,F		+	+	+		bulbar palsy	GBS	n.d.	n.d.	IVIg, PP	Oxford–AstraZeneca	12 days	[9]
19	69,F		+	+		+	bulbar palsy	GBS	n.d.	n.d.	IVIg, MV	Oxford–AstraZeneca	13 days	[9]
20	60,F		+		+		back and legs pain, headache, vomiting	GBS	negative	migraine	IVIg	Johnson & Johnson	10 days	[10]
21	59,M		+	+			postural instability	GBS	negative	HTN, hyperuricemia	IVIg	Oxford–AstraZeneca	10 days	[11]
22	37,M	+	+				back pain	GBS	n.d.	nothing	IVIg	Oxford–AstraZeneca	14 days	[12]
23	73,M	+						GBS	n.d.	HTN, RA	IVIg	Pfizer/BioNTech	16 ~ 17 days	[4]
24	82,F		+				difficulty walking	GBS	n.d.	nothing	IVIg	Pfizer/BioNTech	7 days	[13]
25	71,M				+		headache, ocular pain, ptosis, ataxia	MFS	negative	DM	IVIg	Pfizer/BioNTech	18 days	our case

*GBS* Guillain-Barre syndrome, *MFS* Miller Fisher syndrome, *COVID-19* Coronavirus disease 2019, *FD* Facial diplegia, *OP* Ophthalmoplegia, *RF* Respiratory failure, *AGA* Anti-ganglioside antibodies, *GM3* Anti-GM3 antibody, *AIDP* Acute inflammatory demyelinating polyneuropathy, *BFP* Bifacial weakness with paraesthesias, *ITP* Immune thrombocytopenia, *HTN* Hypertension, *DM* Diabetes mellitus, *RA* Rheumatoid arthritis, *HL* Hyperlipidemia, *n.d*. No data, *MV* Mechanical ventilation, *PP* Plasmaphersis, *PSL* Prednisolone

In the 25 cases studied, including the patient in this case report, the mean age was 58.4 ± 14.7 years (range, 20–82 years). Facial weakness was a frequent symptom observed in 19 patients (76.0%). Four patients (16.0%) presented with ocular motility disorder. Of the 14 cases tested for anti-ganglioside antibodies, only one test positive for it (case 7). This was significantly less than the 50–60% anti-ganglioside antibody positivity recorded in GBS [14] and 80–90% GQ1b positivity in MFS reported in previous reports [15]. One of the cases had a history of GBS (Case 10). Finsterer has mentioned that “Neurologists should remain vigilant for a potential recurrence of GBS after vaccination with a vector-based COVID–19 vaccine” [6]. All authors concluded that “vaccines and GBS may be related, but further research is needed to establish a relationship.” One of the reasons why proving the causal relationship between vaccines and GBS or MFS is difficult is that under the lockdown during the COVID-19 pandemic, the epidemic situation of many infectious diseases causing the antecedent infection of GBS or MFS is also changing. Therefore, this does not justify a negative causal relationship, if GBS or MFS did not increase from baseline after the introduction of COVID-19 vaccines. The most common type of vaccine was Oxford–AstraZeneca. It is possible that this is due to the nature of the vaccine, which is a vector-based vaccine, but it cannot be denied that it may be biased due to the small number of reporters. Of the 25 cases, the period from vaccination to the onset of neurological symptoms was 7–22 days, similar to the duration from the preceding event to the onset of GBS in previous reports [3].

However, as previously reported by Finsterer, COVID-19 infection itself can trigger GBS or MFS [6], and researchers reporting on post-vaccination GBS have emphasized that “even if the vaccine triggers GBS, it is not a reason to recommend refraining from vaccination.”

Although it is unclear about the pathophysiology of GBS and MFS after COVID-19 vaccine, the report that GBS and MFS developed after COVID-19 infection are also almost negative for anti GM1 or GQ1b antibodies may helpful [16]. In other words, if both have the same pathophysiology that is not related to anti-ganglioside antibodies, it is suggested that the vaccine adjuvant and additives are not the cause of the onset but the immune response to the constituent proteins of the virus.

COVID-19 vaccination might have been responsible for the development of MFS in the patient in this case report because of the following reasons: (1) The period from vaccination to neurological symptoms coincided with the time taken for the immune response to occur, (2) neurological symptoms worsened after the second vaccination, and (3) the immunotherapy administered was effective.

Some limitations of our study should be acknowledged. First, facial nerve conduction test and Blink reflex test were not performed, because patient’s consent was not obtained. Second, it is unclear whether the condition of this case is the same as that of classical MFS because anti-GQ1b antibody was negative. In addition, there was a possibility that this patient suffered from the brainstem disorder such as wall-eyed bilateral internuclear ophthalmoplegia (WEBINO) syndrome [17]. Although brainstem disorder was considered, repeated MRI scans including thin-slice brainstem evaluation of the brain were performed, showed no significant lesion.

## Conclusion

We report the first case of COVID-19 vaccination-associated MFS. However, it is difficult to deny that this result may be a coincidence in time, and therefore, no cause-and-effect relationship can be concluded at this time. However, it is indeed true that this report raises an important and timely issue. Furthermore, note that additional research is needed to establish an association between MFS and COVID-19 vaccination. We want to highlight that the risk of neurological complications or any other adverse effects associated with COVID-19 vaccination is low and the benefits of the vaccination outweigh any potential risks or side effects at both individual and social levels. However, as a clinical practice, we currently intend to highlight a precaution in patients with a history of MFS or GBS after COVID-19 vaccination for revaccination (i.e., the second dose). We support and encourage the recommendations of the Centers for Disease Control and Prevention and the guidelines by the World Health Organization for COVID-19 vaccination.

## Data Availability

All data generated or analysed during this study are included in this published article.

## Abbreviations

COVID-19: The coronavirus disease 2019
SARS-CoV-2: severe acute respiratory syndrome coronavirus 2
GBS: Guillain–Barré syndrome
MFS: Miller Fisher syndrome
CSF: cerebrospinal fluid
MRI: magnetic resonance imaging
PET/CT: positron emission tomography/computed tomography
IVIg: intravenous immunoglobulin
WEBINO syndrome: wall-eyed bilateral internuclear ophthalmoplegia syndrome
